# Social capital building interventions and self-reported post-disaster recovery in Ofunato, Japan

**DOI:** 10.1038/s41598-022-14537-8

**Published:** 2022-06-17

**Authors:** Juheon Lee, Daniel P. Aldrich, Emi Kiyota, Tanaka Yasuhiro, Yasuyuki Sawada

**Affiliations:** 1 Midwestern State University, 3410 Taft Boulevard, O’Donohoe Hall 203, Wichita Falls, TX 76308 USA; 2 Northeastern University, 215H Renaissance Park, 360 Huntington Avenue, Boston, MA 02115 USA; 3 Ibasho, 2519 Connecticut Ave NW, Washington, DC 20008 USA; 4 The University of Tokyo, 7-3-1 Hongo, Bunkyo-ku, Tokyo, 113-0033 Japan

**Keywords:** Environmental social sciences, Environmental impact, Natural hazards

## Abstract

Evidence shows that communal resources, cohesion, and social infrastructure can mitigate shocks and enhance resilience. However, we know less about how specific social capital building interventions facilitate recovery in post-disaster environments. Using a survey of over 1000 residents of Ofunato, Japan after the 2011 Tohoku earthquake and tsunami, this study demonstrates that the individuals who actively participated in a community center—created for and led by neighborhood elders—reported higher levels of family and neighborhood recovery than similar individuals who did not participate. Results from ordinal logistic regression analyses, propensity score matching (PSM) and coarsened exact matching (CEM) show arguably stronger causal links between bottom-up, microlocal programs to boost connections in post-disaster areas and post-disaster outcomes. Community-based programs that strengthen social ties even among elderly residents can measurably improve their recoveries.

## Introduction

Elderly residents—people aged 60 years and older—are more vulnerable during a disaster than any other age group due to their low mobility, pre-existing disabilities, and lack of access to family care and institutional supports^1–4^. After disasters, they face additional challenges because they are more likely to suffer from post-traumatic stress disorder and physical injuries and less likely to receive loans for reconstruction^5,6^. As many older individuals live alone with fewer social contacts, diminished social networks intensify these age-related vulnerabilities^7^.

Given that deeper reservoirs of social capital—the ties that bind us—can mitigate some of the consequences of disaster shocks, at least partly^8,9^, some communities have started experimenting with bottom-up, tie-building interventions to deliberately create cohesion^10,11^. Among them is the Ibasho project, an elder-led, community-based framework initiated in Ofunato, a city in Japan’s Tohoku region where residents were hit by the 2011 earthquake, tsunami, and nuclear meltdowns, also known as the 3/11 triple disaster (Ibasho homepage: https://ibasho.org/projects/japan). Beginning in 2013, Ibasho has provided a meeting place for dozens of local, elderly citizens through its open floorplan physical facility which houses the Ibasho Café along with other events such as home maintenance assistance, a library, and homework help for students. Ibasho challenged traditional perceptions of aging and helped elders be seen as valuable members of their community^12^.

While these initiatives clearly demonstrate the potential of specific social capital building interventions in accelerating disaster recovery, rigorous quantitative assessments have been largely missing in the existing literature. We aim at bridging this lacuna by uncovering the degree to which participation in the community impacts disaster recovery. In so doing, our article contributes to the literature on social capital, interventions, and recovery in several ways. First, earlier studies found that elderly residents who engaged in bottom-up, community-based programs displayed measurably improved levels of social capital, that is, they developed broader social networks, a deeper sense of belonging, and a deeper sense of efficacy^12,13^. These studies, however, did not seek to go beyond the improved social capital and measure the programs’ impact on post-crisis recovery. This paper does so, illuminating how Ibasho participation—controlling for several other factors—impacted recovery, which scholars have long identified as a poorly understood phenomenon^14,15^.

Second, this article utilizes the residents’ Ibasho visits as well as other social capital indicators—such as participation in voluntary organizations, maintenance of personal network, and trust in community members—to investigate recovery in their families and neighborhoods after the 2011 disaster, controlling for several potential confounding factors. Although this study is not designed for measuring how Ibasho visits are associated with other social capital indicators, our research recognizes that social capital needs to be measured through different instruments in its study of recovery^16^.

Third, we adopt quasi-experimental methods to illuminate a potential causal link running from the intervention to trust in community members. Specifically, our quantitative analysis steps beyond the limitations of standard linear regression, using propensity score matching (PSM) and coarsened exact matching (CEM) techniques^17,18^. In doing so we are better positioned to make arguments not just about patterns in the recoveries of individuals in post-disaster communities, but also about the precise mechanisms through which those events occur.

Finally, the findings of this study can contribute to the broader discussion on global disaster risk reduction (e.g., the Sendai Framework) by providing insights into how local government and non-governmental organizations can intervene and mobilize vulnerable groups of people to build more resilient communities against disasters.

## Age, community social capital, and disaster recovery

Disaster studies have long emphasized that older individuals are more likely to perish, be injured, and suffer from impoverishment and emotional trauma during and after a disaster^1,19^. Chronological age correlates with declines in the ability to adapt disasters in preparation, response, and recovery. Older individuals commonly have impaired vision and hearing, as well as reduced cognition and memory that affect their perception and comprehension of hazard-related warnings^20^. They also display difficulty in performing personal care and travel during a disaster due to their reduced motor strength, impaired balance, and other pre-existing physical conditions^21^.

Due to these physical and cognitive limitations, many elderly citizens are reluctant to leave their homes during shocks^22^. Hence, these limitations make the elderly vulnerable especially during a rapid-onset disaster, such as tornados, earthquakes and floods, which requires speedy evacuation. Even after a disaster, older individuals tend to face precarity in their health situations especially when because pre-existing conditions (diabetes, cancer, etc.) may be harder to treat after the disaster^6^.

Beyond the disaster victims’ age, studies have emphasized that elderly residents face socio-economic changes related to age, as their vulnerability comes more from socioeconomic changes than age^7^. Due to their limited sources of income, older people are less likely to engage in preparation activities such as purchasing disaster equipment or insurance that may be critical to their survival and recovery^2,5^. In many cases, elderly survivors have not qualified for loans due to their lack of employment and fail to receive proper information about government aid programs, slowing their economic recovery^5^. Psychologically, many older individuals are hesitant to apply for aid due to their fear of stigma or other unfounded concerns about receiving assistance^21^.

A more serious problem, however, comes from reduced social connections. Many elderly individuals live alone with limited social contacts with their family, friends, and neighbors, which makes them under-utilize social capital that is critical to their survival and wellbeing during an emergency^7^. Social capital, therefore, is particularly crucial to the elderly citizens. For example, following the 2011 earthquake, tsunami, and nuclear meltdowns in Tohoku, Japan many elderly people were not able to evacuate to shelters immediately after the earthquake due to their low mobility and lack of information. Those who received help from their neighbors, family, and from the governmental institutions, however, were able to evacuate and survive^23^. Similarly, after the Brisbane floods in 2011 and 2013, elderly survivors said that they relied on their bonding networks and the assistance from community members that they felt were crucial to their recovery^19,24^.

Given the importance of social capital for elderly citizens, locals created the Ibasho project based on the belief that an elder-led, community-based framework can empower the elderly, build cohesion, and develop community resilience against future crises^12^. After the 3/11 triple disasters in Tohoku, many of those whose homes were destroyed evacuated to temporary shelters with limited privacy^25^. Ibasho provided a space where survivors could socialize freely outside their shelter homes (similar to the temporary housing provided by the US Federal Emergency Management Agency). The Ibasho Café held regular meetings for community elders and provided community meetings that included residents of all ages including children, young mothers, and other elderly citizens.

While earlier studies demonstrated the possibility of social capital building, they did not examine how Ibasho visits and the residents’ reservoirs of social capital correlate with their post-disaster recovery^13^. Given a recognized, positive relationship between social capital and post-disaster recovery, we hypothesized that greater participation in the Ibasho project would correlate with higher degrees of recovery^26,27^. Previous studies have demonstrated that communities with deeper reservoirs of social capital make residents trust each other, grow a strong sense of belonging, and create more cohesive networks, making the community more resilient. In this study, we test this hypothesis through large-N statistical modeling that includes the Rubin causality model of program evaluation. We quantify the causal impact of the Ibasho project by defining the “treatment” group as those who had visited the Ibasho Café and the “control” group as those who has not visited there.

In addition to the effect of Ibasho visits, this study tests three widely used measures of social capital: residents’ participation in voluntary organizations, number of friends, and trust in their community members. Measuring social capital has been one of the key questions among scholars; however, Aldrich and Meyer distinguished between cognitive approaches and behavioral approaches that are useful for studying disaster resilience^16^. Studies that adopted a cognitive approach have focused on people's level of trust in others as the core of social capital and disaster resilience; while studies that focused on behavioral manifestation of social capital utilized data on residents’ participation in voluntary organizations such as religious, civic, or political organizations, that are related to disaster preparedness and resilience^28–32^. Social networks also serve as important indicators of behavioral social capital; studies have used survey questions to ask the respondents’ number of friends and contacts so that they can discuss their problems^28^. These behavioral and cognitive approaches are used to properly examine different aspects of social capital or compare disaster resilience of different regions^33^.

## Methods

### Data collection

Several local social welfare and educational organizations in the city of Ofunato supported the collection of data used for this analysis. Volunteers from the Masaaki elementary and middle schools, temporary housing facilities (*kasetsu jūtaku*), a local hospital, and the municipal offices carried out the survey. After two rounds of pilot tests, one in early 2013 to refine the survey questions and the other in the summer of 2013 before the start of Ibasho facility, enumerators carried out a survey of 1142 respondents in the fall of 2014, more than one year after the opening of the Ibasho Café^13^. The volunteers sampled ten neighborhoods across Ofunato city, each of which had 1000–7500 residents. We used geographic-based randomized sampling to ensured that the survey included those who live in 10 neighborhood where the Ibasho facility is located (i.e., Massaki-cho) and those living in the other 9 neighborhoods of Ofunato city. The respondents’ residential areas were coded by neighborhood to be included in regression models. The survey was carried out in accordance with relevant IRB guidelines and regulations (Northeastern University #15-10-33).

### Variables

Self-evaluated recovery progress in their homes and communities serves as the core dependent variable in this study. The survey asked the respondents two questions about recovery: “Would you say that the recovery/reconstruction efforts for your family have made progress?” (Question 15); and “Would you say that the recovery/reconstruction efforts for our neighborhood have made progress?” (Question 16). The responses were coded using a five-point Likert scale: great progress, some progress, couldn’t say, not so much progress, and no progress. Although these variables do not capture objective progress of recovery (such as physical infrastructure recovery, the reopening of medical facilities, etc.), past studies regularly relied on individuals’ subjective evaluations including self-rating of their physical and mental health conditions and their satisfaction with recovery programs^28,34,35^.

Among the independent variables, the survey asked respondents how often they visited the Ibasho house (Question 28–29). Their answers were coded on a seven-point Likert scale (0–6): daily, 3–4 times a week, once a week, a few times a month, once a month, less than once a month, and never visited. In addition, the survey included three social capital indicators. For participation in voluntary organizations, the survey asked if they are a member of any organization, such as unions, cooperative organizations, volunteer groups, senior clubs, wives clubs, or some similar groups (Question 10). The answers were binary (yes/no), therefore, the responses were coded in a binary fashion. For personal networks, the survey asked how many people in their neighborhood they talked with regularly (Question 19). The suggested answers were: none, 1 person, several people, 10 people, and 50 people or more. The responses were coded using 5-point Likert scale (0–4). For the respondents’ trust, the survey asked how much they trust the people in their neighborhood. Three possible answers were suggested: yes, not sure, and no (0–2).

Control variables included respondents’ age, gender, duration of residence, education, and sources of income. Age was coded using a 5-point Likert scale: 18–29, 30–44, 45–59, 60–74, 75 and above. Education was coded as: elementary school, middle school, high school, colleges of technology, junior colleges, universities, and post-graduate programs. Regarding the respondents’ income, too many respondents chose not to report their income; therefore, income was measured by the number of sources rather than the total amount: none; 1 source among salary, pension, and self-employed; and multiple sources.

Table 1 provides descriptive statistics for all variables in the analysis. Descriptive statistics are reported separately for the treatment group (those who had visited the Ibasho Café) and the control group (those who had not visited the Ibasho Café) because the two groups need to be distinguished for propensity score matching (PSM) and coarsened exact matching (CEM) analyses. As the table shows, most of the respondents had not visited the Ibasho Café, which provides a robust comparison group for counterfactuals.

**Table 1:** Descriptive statistics for variables.

Variables	Pooled			Ibasho Visits = 1 (treatment)		Ibasho Visit = 0 (control)		Baseline balance (treatment minus control)
	Min–Max	N	Mean (SD)	N	Mean (SD)	N	Mean (SD)	Mean (SD)
**Family and community resilience**								
Family recovery progress	0–4	1143	2.59 (1.21)	114	2.81 (1.10)	1030	2.57 (1.22)	0.24 (− 0.12)
Neighborhood recovery progress	0–4	1143	2.51 (1.13)	114	2.57 (1.08)	1030	2.50 (1.14)	0.07 (− 0.06)
Ibasho visits	0–6	1143	0.18 (0.69)	114	1.78 (1.39)	1030	0 (0)	1.78 (1.39)
**Social capital indicators**								
Participation in voluntary organizations	0–1	1143	0.57 (0.50)	114	0.70 (0.46)	1030	0.55 (0.50)	0.15 (− 0.04)
Personal network (people you can talk to)	0–4	1143	2.89 (0.93)	114	3.10 (0.85)	1030	2.87 (0.94)	0.23 (− 0.09)
Trust (in people of neighborhood)	0–2	1143	1.56 (0.48)	114	1.71 (0.56)	1030	1.54 (0.62)	0.17 (− 0.06)
**Demographic factors**								
Age	1–5	1143	2.73 (1.09)	114	3.06 (1.03)	1030	2.70 (1.09)	0.36 (− 0.06)
Gender (male = 0/female = 1)	0–1	1143	0.44 (0.50)	114	0.38 (0.49)	1030	0.45 (0.50)	− 0.07 (0.01)
Duration of residence (years)	0.1–100	1143	26.71 (21.98)	114	32.00 (23.22)	1030	26.11 (21.77)	5.89 (1.45)
Education	1–8	1143	4.02 (1.38)	114	4.17 (1.42)	1030	4.00 (1.37)	0.17 (0.05)
Income source (0–2)	0–2	1143	1.07 (0.29)	114	1.08 (0.27)	1030	1.07 (0.30)	0.01 (− 0.03)

### Regression analysis

As the two dependent variables are based on the ordinal scale questions, we ran ordinal logistic regression models (reported in Tables 3 and 4). As ordinal logistic regression assumes that independent variables are proportional across the different thresholds of the dependent variable (i.e., the proportional odds assumption), we implemented the Brant test and found that the proportional odds assumption holds for all regression models^36^. The regression models were structured hierarchically to determine whether adding an independent variable better explained the overall variance of the output variable. To rule out unknown effects of regional differences, we used fixed effects by adding dummy variables for the 10 neighborhoods of Ofunato city. Moreover, to manage heteroscedasticity, standard errors were clustered by neighborhood. For all regression models, the variance inflation factor for all models was below 3.0, which is accepted by most social science researchers.

As this study uses a single-year survey dataset, endogeneity can be a potential problem. Therefore, two additional models were structured using two quasi-experimental methods: propensity score matching (PSM) and coarsened exact matching (CEM). Propensity score matching addresses the selection bias problem through propensity scores^17^. This study used the Inverse Probability Weighting method to avoid a smaller sample size^37^. First, a propensity score for each respondent was calculated using logistic regression, with a logistic regression determining the probability of the treatment group or control group. Then the propensity score was transformed to provide different weights to the treatment group and control group^38^. When different weights are given based on the propensity score, the resulting groups have similar characteristics to randomly assigned groups in an experiment^39^. Table 2 reports the results of covariate balance tests. The balance for each covariate was tested by regressing each covariate on treatment (Ibasho visit = 1/0) [for details, see Ref.^38^]. Covariates were reported as unbalanced if the coefficients were statistically significant (p < 0.05) and balanced if the coefficients were not significant (p > 0.05). As the table shows, propensity score weighting improved the balance for most variables, but neighborhood recovery progress, age, and income source variables became or remained unbalanced after weighting. In addition to the PSM, we also carried out coarsened exact matching (CEM), as some scholars have criticized PSM of its bias and inefficiency^18^. For CEM, all observations are first temporarily coarsened and sorted into different bins based on the chosen variables (i.e., independent variables). Matching individuals are then found based on these bins; others are discarded. After having a new treatment group and a control group using PSM and CEM methods, we used ordinal logistic regression to report the results.

**Table 2:** Balance assessment for propensity score weighting.

Covariates	Unweighted	Propensity score weighted
Family recovery progress	Balanced (0.1 < p < 1)	Balanced (0.1 < p < 1)
Neighborhood recovery progress	Balanced (0.1 < p < 1)	Unbalanced (0.001 < p < 0.01)
Participation in voluntary organizations	Unbalanced (0.01 < p < 0.05)	Balanced (0.1 < p < 1)
Personal network	Unbalanced (0.01 < p < 0.05)	Balanced (0.1 < p < 1)
Trust	Unbalanced (0.01 < p < 0.05)	Balanced (0.1 < p < 1)
Age	Unbalanced (0 < p < 0.001)	Unbalanced (0.001 < p < 0.01)
Gender	Unbalanced (0 < p < 0.001)	Balanced (0.1 < p < 1)
Duration of residence	Unbalanced (0.01 < p < 0.05)	Balanced (0.1 < p < 1)
Education	Balanced (0.1 < p < 1)	Balanced (0.1 < p < 1)
Income source	Balanced (0.1 < p < 1)	Unbalanced (0.01 < p < 0.05)

### Approval for human experiments

The study was carried out in accordance with relevant Institutional Review Board guidelines and regulations of Northeastern University (Approval number: #15-10-33/Committee: C. Randall Colvin and Nan C. Regina). All subjects are over 18, and informed consent was obtained from all subjects. The study, including all experimental protocols, was approved by Institutional Review Board of Northeastern University.

## Results

As the first step, the respondents’ self-evaluated recovery progress of their family was regressed using their Ibasho visits and the three social capital indicators. In Table 3, five regression models (Models 1–5) were reported in a hierarchical way to determine whether adding an independent variable contributed to the model that explains the overall variance of the output variable. Overall, the gradual decrease of residual deviance and Akaike Information Criterion values from model 1 to model 5 indicates that each independent variable contributed to the explanatory power of the models and are useful predictors of the recovery progress of families. Two additional models were structured for PSM (Model 6) and CEM (Model 7) analysis.

**Table 3:** Regression of family recovery progress on Ibasho visits and social capital indicators.

Family recovery progress	Ordinal logistic regression					PSM	CEM
	(1)	(2)	(3)	(4)	(5)	(6)	(7)
Ibasho visits					0.167 [1.182] (0.052)***	0.220 [1.246] (0.051)***	0.165 [1.180] (0.080)**
**Social capital indicators**							
Participation in voluntary organizations				0.239 [1.270] (0.146)	0.233 [1.262] (0.143)	0.289 [1.334] (0.121)**	0.296 [1.345] (0.207)
Personal network			0.267 [1.306] (0.061)***	0.237 [1.267] (0.071)***	0.240 [1.271] (0.073)***	0.543 [1.720] (0.066)***	0.205 [1.228] (0.085)**
Trust		0.567 [1.763] (0.154)***	0.487 [1.628] (0.163)***	0.463 [1.589] (0.158)***	0.462 [1.588] (0.157)***	0.329 [1.389] (0.091)***	0.445 [1.561] (0.183)**
**Control variables**							
Age	− 0.218 [0.804] (0.048)***	− 0.256 [0.774] (0.056)***	− 0.272 [0.761] (0.060)***	− 0.277 [0.757] (0.060)***	− 0.306 [0.736] (0.061)***	− 0.190 [0.826] (0.061)***	− 0.282 [0.753] (0.161)**
Gender (M = 0/F = 1)	0.018 [1.018] (0.179)	0.009 [1.009] (0.187)	− 0.013 [0.987] (0.197)	− 0.012 [0.988] (0.191)	− 0.011 [0.988] (0.192)	− 0.284 [0.752] (0.101)**	0.028 [1.028] (0.190)
Duration of residence	− 0.000 [0.999] (0.006)	− 0.002 [0.997] (0.007)	− 0.006 [0.994] (0.007)	− 0.007 [0.992] (0.007)	− 0.007 [0.993] (0.007)	− 0.022 [0.978] (0.003)***	− 0.005 [0.994] (0.007)
Education	0.182 [1.200] (0.036)***	0.166 [1.180] (0.035)***	0.167 [1.182] (0.035)***	0.167 [1.182] (0.034)***	0.157 [1.170] (0.037)***	0.222 [1.249] (0.042)***	0.173 [1.189] (0.067)***
Sources of Income	0.303 [1.354] (0.272)	0.269 [1.309] (0.273)	0.235 [1.265] (0.226)	0.231 [1.260] (0.266)	0.223 [1.250] (0.262)	0.753 [2.123] (0.206)***	0.153 [1.165] (0.284)
**Neighborhood Dummies (Reference: Massaki area)**							
Sakari area	0.030 [1.030] (0.027)	0.104 [1.109] (0.104)	0.190 [1.209] (0.117)	0.177 [1.194] (0.117)	0.276 [1.317] (0.122)**	− 0.249 [0.779] (0.199)	0.576 [1.780] (0.115)***
Ofunato area	− 0.351 [0.703] (0.064)***	− 0.238 [0.787] (0.075)***	− 0.138 [0.871] (0.076)*	− 0.120 [0.886] (0.082)	− 0.020 [0.980] (0.092)	0.113 [1.119] (0.164)	0.223 [1.250] (0.081)***
Akasaki area	− 0.538 [0.583] (0.035)***	− 0.505 [0.603] (0.034)***	− 0.442 [0.642] (0.038)***	− 0.428 [0.651] (0.043)***	− 0.321 [0.725] (0.053)***	− 0.752 [0.471] (0.162)***	− 0.220 [0.801] (0.061)***
Ikawa area	− 0.361 [0.696] (0.131)***	− 0.226 [0.766] (0.139)*	− 0.179 [0.836] (0.153)	− 0.154 [0.857] (0.158)	− 0.048 [0.952] (0.163)	− 0.031 [0.969] (0.164)	0.039 [1.040] (0.159)
Takkon area	− 0.375 [0.686] (0.127)***	− 0.340 [0.711] (0.136)**	− 0.211 [0.809] (0.147)	− 0.204 [0.815] (0.150)	− 0.104 [0.900] (0.158)	− 0.029 [0.971] (0.217)	0.097 [1.102] (0.152)
Hikoroichi area	− 0.774 [0.461] (0.060)***	− 0.888 [0.411] (0.084)***	− 0.907 [0.403] (0.087)***	− 0.909 [0.402] (0.086)***	− 0.817 [0.441] (0.077)***	− 0.152 [0.858] (0.276)	− 0.836 [0.433] (0.095)***
Sanrikucho-Ryori area	0.119 [1.126] (0.036)***	0.103 [1.108] (0.043)**	0.107 [1.113] (0.048)**	0.145 [1.157] (0.052)***	0.243 [1.275] (0.051)***	− 1.034 [0.355] (0.199)***	0.228 [1.257] (0.060)***
Sanrikucho-Okirai area	− 0.384 [0.681] (0.029)***	− 0.376 [0.686] (0.030)***	− 0.306 [0.736] (0.037)***	− 0.284 [0.752] (0.032)***	− 0.170 [0.843] (0.047)***	− 0.945 [0.388] (0.347)***	− 0.376 [0.686] (0.062)***
Sanrikucho-Yoshihama area	0.121 [1.129] (0.043)***	0.161 [1.175] (0.037)***	0.181 [1.197] (0.044)***	0.174 [1.190] (0.045)***	0.275 [1.316] (0.043)***	1.484 [4.409] (0.357)***	0.369 [1.447] (0.085)***
**Intercepts**							
1	− 2.356*** (0.192)	− 1.741*** (0.228)	− 1.229*** (0.207)	− 1.266*** (0.209)	− 1.270*** (0.222)	− 0.678 (0.509)	− 1.566** (0.632)
2	− 1.572*** (0.238)	− 0.946*** (0.272)	− 0.430 (0.280)	− 0.464* (0.276)	− 0.466* (0.257)	0.874 (1.831)	− 0.493 (0.745)
3	− 0.117 (0.231)	0.528** (0.250)	1.061*** (0.281)	1.031*** (0.274)	1.034*** (0.269)	2.442 (2.035)	1.091 (0.735)
4	1.205*** (0.266)	1.882*** (0.227)	2.427*** (0.276)	2.399*** (0.275)	2.404*** (0.254)	3.702* (2.072)	2.510*** (0.684)
Observations	770	770	770	770	770	770	560
Residual Deviance	2181.242	2156.22	2146.02	2143.57	2140.86	4405.354	1514.303
Akaike Information Criterion	2217.242	2194.22	2186.02	2185.57	2184.86	4449.354	1558.303

*p < 0.1; **p < 0.05; ***p < 0.01; Odds ratios are reported in square brackets; standard errors (in parentheses) are clustered by neighborhood.

The coefficient of Ibasho visits in Model 5 indicated that, for a one-unit increase in Ibasho visits, the expected change of family recovery in log odds is 0.167 (p < 0.01). The odds ratio of 1.182 (exponentiated value of 0.167) indicates that the respondents’ Ibasho visits increase the odds of family recovery progress by 18.2%. This means that the respondents who had visited Ibasho Café more frequently tended to see greater progress in the recovery and reconstruction of their families than those who had not. The significance of Ibasho visits was also found in the PSM model in Model 6 (B = 0.220, OR 1.246, p < 0.01) and CEM model in Model 7 (B = 0.165, OR 1.180, p < 0.05), which means that the respondents’ Ibasho visits increased the probability of higher degree of recovery in their families.

Three social capital indicators generally showed positive associations with family recovery progress as well. People who had membership in a voluntary organization showed greater progress in their family recovery, which was shown in Model 6 (B = 0.289, OR 1.334, p < 0.05); however, the results did not show statistical significance in other models. Personal networks displayed a strong positive relationship with family recovery progress, shown in Model 5 (B = 0.240, OR 1.271, p < 0.01), and the result was supported by the PSM and CEM models in Model 6 (B = 0.543, OR 1.720, p < 0.01) and Model 7 (B = 0.205, OR 1.228, p < 0.05). The respondents’ trust in people of neighborhood also showed a positive relationship with their family recovery progress, demonstrated in Model 5 (B = 0.462, OR 1.588, p < 0.01), as well as in Model 6 (B = 0.329, OR 1.389, p < 0.01) and Model 7 (B = 0.445, OR 1.561, p < 0.01), which supported our hypothesis.

Among the demographic factors, age and education showed strong associations with family recovery progress across all models. Age in Model 5 demonstrated a negative association with family recovery progress (B = -0.306, OR 0.736, p < 0.01), which means that older respondents tended not to see expected progress in post-disaster recovery in their families. This result was supported by PSM and CEM models and with the theories discussed above about the impact age has on disaster survivors. On the contrary, education in Model 5 showed a positive association with family recovery progress (B = 0.157, OR 1.170, p < 0.01), meaning that highly educated people tend to see greater recovery in their families. The result was consistent in PSM and CEM models. Gender, duration of residence, and sources of income did not show consistent results with statistical significance. However, PSM model (Model 6) showed negative effects of gender (B = − 0.284, OR 0.752, p < 0.05) and duration of residence (B = − 0.022, OR 0.978, p < 0.01) on family recovery progress, which means that female residents and long-time residents may be less likely to be satisfied with recovery in their families.

Neighborhood dummy variables showed a pattern that invites further studies. Most coefficients were negative in reference to Massaki area, which means that Massaki residents generally highly evaluate their family recovery progress compared to other areas. The exception is two coastal neighborhoods (Sanrikucho-Ryori area and Sanrikucho-Yoshihama area) that showed a higher level of family recovery progress than Massaki area where Ibasho Café is located. As regional comparison is not a focus of this study, further studies should be conducted to explain this high family recovery progress in these two neighborhoods.

Next, the respondents’ evaluation of their neighborhood recovery progress was regressed on their Ibasho visits and other social capital indicators, which is illustrated in Table 4. Again, five ordinal logistic regression models were structured hierarchically, and fixed effects were used for ten neighborhoods. To manage heteroscedasticity, we clustered standard errors by neighborhood. Again, the gradual decrease of residual deviance and Akaike Information Criterion values from model 1 to model 5 showed that all independent variables contribute to the explanatory power of the models and are useful indicators for neighborhood recovery progress.

**Table 4:** Regression of neighborhood recovery progress on Ibasho visits and social capital indicators.

Neighborhood recovery progress	Ordinal logistic regression					PSM	CEM
	(1)	(2)	(3)	(4)	(5)	(6)	(7)
Ibasho visits					0.130 [1.139] (0.042)***	0.039 [1.039] (0.051)	0.113 [1.120] (0.065)**
**Social capital indicators**							
Participation in voluntary organizations				0.223 [1.249] (0.106)**	0.221 [1.247] (0.107)**	0.244 [1.276] (0.127)*	0.281 [1.325] (0.167)*
Personal network			0.057 [1.059] (0.078)	0.029 [1.029] (0.079)	0.029 [1.030] (0.079)	− 0.068 [0.933] (0.065)	− 0.046 [0.954] (0.079)
Trust		0.569 [1.767] (0.099)***	0.552 [1.737] (0.101)***	0.531 [1.701] (0.103)***	0.530 [1.701] (0.102)***	0.471[1.601] (0.097)***	0.546 [1.727] (0.214)**
**Control variables**							
Age	− 0.148 [0.862] (0.041)***	− 0.182 [0.833] (0.047)***	− 0.186 [0.829] (0.049)***	− 0.192 [0.825] (0.053)***	− 0.214 [0.806] (0.056)***	0.254 [1.289] (0.064)***	− 0.105 [0.900] (0.120)
Gender (M = 0/F = 1)	0.055 [1.057] (0.173)	0.048 [1.049] (0.177)	0.044 [1.045] (0.179)	0.044 [1.045] (0.176)	0.046 [1.047] (0.174)	0.215 [1.239] (0.104)**	0.198 [1.219] (0.216)
Duration of residence	− 0.001 [0.998] (0.002)	− 0.004 [0.995] (0.004)	− 0.005 [0.994] (0.004)	0.006 [0.993] (0.004)	− 0.006 [0.993] (0.004)	− 0.024 [0.976] (0.004)***	− 0.008 [0.991] (0.005)
Education	0.153 [1.165] (0.050)***	0.141 [1.152] (0.047)***	0.140 [1.151] (0.046)***	0.138 [1.148] (0.046)***	0.129 [1.138] (0.043)***	0.319 [1.375] (0.044)***	0.135 [1.145] (0.030)***
Sources of income	0.310 [1.364] (0.152)**	0.262 [1.299] (0.166)	0.255 [1.290] (0.166)	0.255 [1.290] (0.164)	0.244 [1.276] (0.170)	0.936 [2.550] (0.214)***	0.390 [1.477] (0.247)
**Neighborhood dummies (Reference: Massaki area)**							
Sakari area	0.406 [1.501] (0.076)***	0.495 [1.641] (0.098)***	0.514 [1.672] (0.103)***	0.501 [1.651] (0.105)***	0.583 [1.792] (0.115)***	0.418 [1.519] (0.214)*	0.243 [1.276] (0.108)**
Ofunato area	0.182 [1.200] (0.036)***	0.280 [1.324] (0.057)***	0.304 [1.355] (0.070)***	0.320 [1.378] (0.071)***	0.409 [1.505] (0.095)***	0.088 [1.092] (0.168)	0.604 [1.829] (0.105)***
Akasaki area	− 1.128 [0.323] (0.045)***	− 1.084 [0.338] (0.048)***	− 1.072 [0.342] (0.055)***	− 1.065 [0.344] (0.054)***	− 0.976 [0.376] (0.069)***	− 2.028 [0.131] (0.167)***	− 1.141 [0.319] (0.099)***
Ikawa area	0.116 [1.123] (0.081)	0.182 [1.199] (0.103)*	0.201 [1.223] (0.112)*	0.233 [1.262] (0.114)**	0.320 [1.377] (0.130)**	− 0.432 [0.648] (0.168)**	0.352 [1.422] (0.153)**
Takkon area	0.744 [2.104] (0.088)***	0.801 [2.227] (0.110)***	0.829 [2.292] (0.119)***	0.842 [2.322] (0.119)***	0.925 [2.522] (0.133)***	2.025 [7.577] (0.246)***	1.233 [3.433] (0.174)***
Hikoroichi area	− 1.004 [0.366] (0.028)***	− 1.141 [0.319] (0.034)***	− 1.144 [0.318] (0.034)***	− 1.153 [0.315] (0.035)***	− 1.078 [0.340] (0.035)***	− 0.438 [0.645] (0.264)*	− 1.075 [0.341] (0.080)***
Sanrikucho-Ryori area	0.534 [1.706] (0.036)***	0.490 [1.633] (0.035)***	0.492 [1.636] (0.032)***	0.531 [1.700] (0.035)***	0.617 [1.854] (0.035)***	− 0.585 [0.557] (0.225)***	0.564 [1.758] (0.057)***
Sanrikucho-Okirai area	0.527 [1.694] (0.023)***	0.589 [1.803] (0.025)***	0.604 [1.831] (0.028)***	0.637 [1.891] (0.037)***	0.735 [2.087] (0.060)***	− 0.304 [0.737] (0.361)	0.457 [1.580] (0.075)***
Sanrikucho-Yoshihama area	1.216 [3.376] (0.066)***	1.238 [3.449] (0.071)***	1.241 [3.460] (0.068)***	1.249 [3.487] (0.069)***	1.331 [3.787] (0.056)***	1.470 [4.347] (0.303)***	1.444 [4.241] (0.051)***
**Constant**							
1	− 2.515*** (0.466)	− 1.914*** (0.518)	− 1.804*** (0.560)	− 1.843*** (0.555)	− 1.856*** (0.527)	− 0.586 (3.787)	− 1.598*** (0.413)
2	− 0.927** (0.377)	− 0.310 (0.444)	− 0.201 (0.494)	− 0.236 (0.487)	− 0.246 (0.440)	1.252 (2.792)	0.055 (0.381)
3	− 0.001 (0.386)	0.628 (0.461)	0.738 (0.519)	0.706 (0.508)	0.699 (0.478)	2.510 (2.899)	0.989** (0.432)
4	2.306*** (0.411)	2.989 (0.507)***	3.101*** (0.539)	3.072*** (0.531)	3.068*** (0.492)	5.125* (3.018)	3.397*** (0.430)
Observations	770	770	770	770	770	770	560
Residual deviance	2043.46	2019.14	2018.67	2016.58	2014.79	4145.95	1441.29
Akaike information criterion	2079.46	2057.14	2058.67	2058.58	2058.79	4189.95	1485.29

*p < 0.1; **p < 0.05; ***p < 0.01; Odds ratios are reported in square brackets; standard errors (in parentheses) are clustered by neighborhood.

The coefficient of Ibasho visits in Model 5 indicated that, for a one-unit increase in Ibasho visits, the expected change of neighborhood recovery in log odds is 0.130. The odds ratio 1.139 (exponentiated value of 0.130) indicates that the respondents’ Ibasho visits increase the odds of neighborhood recovery progress by 13.9%, meaning that the respondents who frequently visit Ibasho tended to see greater progress in their neighborhood recovery. This result was statistically significant (p < 0.01) and was supported by CEM model, shown in Model 7 (B = 0.113, OR 1.120, p < 0.05). The PSM model (Model 6) did not show statistical significance.

Among the three social capital indicators, only trust consistently showed statistically significant relationships with neighborhood recovery progress. Participation in voluntary organization in Model 5 indicated a strong positive relationship with neighborhood recovery progress (B = 0.221, OR 1.247, p < 0.05). PSM model (Model 6) and CEM models only weakly supported the result. On the contrary, respondents’ personal network did not show statistical significance in its relationship with the neighborhood recovery progress across all models, which means that neighborhood recovery is not associated with how individuals have many friends in the neighborhood. However, trust in Model 5 showed that it is strongly associated with neighborhood recovery progress (B = 0.530, OR 1.701, p < 0.01), which was supported by PSM and CEM models in Model 6 (B = 0.471, OR 1.601, p < 0.01) and in Model 7 (B = 0.546, OR 1.727, p < 0.05).

Age was negatively associated with neighborhood recovery progress in Model 1–5, which means that older respondents tended not to see the expected progress in post-disaster recovery in their neighborhoods. However, this relationship was not strongly supported by the CEM model, which questions a causal link between age and neighborhood recovery progress. On the contrary, education showed a positive relationship with family recovery progress across all models. Gender and sources of income did not consistently show statistical significance with neighborhood recovery progress; however, PSM models showed that male respondents and the long-time residents may not be satisfied with their neighborhood recovery progress.

Neighborhood dummy variables also showed a pattern that invites further studies. Most coefficients were positive in reference to Massaki area, which means that Massaki residents generally poorly evaluate their family recovery progress compared to other areas. The exception is two neighborhoods (Akasaki area and Hikoroichi area) that showed a lower level of neighborhood recovery progress than Massaki area where Ibasho Café is located. Again, a comparing neighborhood is not a focus of this study; therefore, further studies should be conducted to explain this relatively lower recovery of Massaki area as well as Akasaki and Hikoroichi neighborhoods.

## Discussion

This study builds upon the previous studies on social capital intervention projects that showed how a local, elder-managed social capital building project could empower the elderly and help them contribute to their community by improving cohesion, efficacy, and sense of place^10,11,13^. Unlike these previous studies, we did not seek to measure how the Ibasho project promotes social capital among the participants. This study aimed at demonstrating how the elder-led community project, together with other social capital indicators, can improve the (self-reported) recovery after the 3/11 triple disasters in Japan. The regression models of this study consistently showed that Ofunato residents who had visited the Ibasho Café frequently over a year made better progress in recovery within their families and neighborhoods compared to those who did not visit. Two quasi-experimental methods were added to address the limitations of a single-year survey dataset, and their results strongly supported the findings.

Together with Ofunato residents’ Ibasho visits, three typical measures of social capital were tested on recovery. The results generally displayed positive relationships between social capital measures and disaster recovery. Respondents who had participated in voluntary organizations, a greater number of friends, and a higher level of trust in the community showed better progress in post-disaster recovery in the family. However, for the perceived neighborhood recovery, only the effect of trust was consistently strong. The effect of participation in the voluntary organization was relatively weak, and personal network (measured by the number of friends) was not significantly associated with neighborhood recovery progress. As the dependent variable is self-evaluation of neighborhood recovery, having many friends may have given the respondents chances to compare with other neighborhoods and lowered their satisfaction. This may be related to the results of neighborhood dummy variables that showed that Massaki residents tend to poorly evaluate their neighborhoods recovery progress.

These findings have implications for elderly citizens, especially considering the rising population of the elderly in Japan (and other advanced industrial democracies). The elderly are traditionally perceived as weak and in need of care during a disaster. All models of this study also proved that respondents’ age is negatively associated with making progress in recovery. However, the participants of Ibasho Café showed that community elders coul be empowered and become an essential resource for community resilience and disaster recovery. The elderly are typically less likely to be employed and engaged in social activities; therefore, encouraging them to engage with a community-building project may draw them out from this potential isolation and help enhance the overall community’s resilience. Programs like Ibasho can mitigate risks and enhance resilience by mobilizing community elders.

These results encourage other nonprofit organizations and disaster managers to rethink the potential role of the elderly in enhancing social ties in their communities. Moreover, first responders and disaster managers should ensure that disaster mitigation measures, such as the rapid but random assignment of survivors to temporary shelters, do not damage existing social networks^40^. Although every aspect of the Japanese case is not directly applicable to other social and economic contexts, it is important to note that the cost of Ibasho program is relatively low compared to technology-driven approaches or physical infrastructure building, which suggest that this type of project can be ideal for other settings, such as developing countries with less material resources, or societies with a growing population of elderly whose experience and wisdom could be of use to younger generations. United Nations Sendai Framework for Disaster Risk Reduction 2015–2030 recognizes increasing mortality and economic losses from disasters especially among the most vulnerable groups in societies and emphasizes that the losses are disproportionately higher in developing countries than in developed countries (see United Nations website: www.undrr.org). The framework, therefore, calls for more active roles of governments and relevant stakeholders in identifying and managing various disaster risks. This study, conducted after the 2011 triple disaster, still provides valuable insights into the roles of local governments and non-governmental organizations in protecting vulnerable groups and building more resilient communities through this elder-led local program.

There are limitations of this study that can be addressed in the future studies. First, while this study relied on the respondents’ self-evaluation of recovery, further studies could seek to draw on objective recovery data. Second, the survey did not distinguish community elders by their levels of participation in the Ibasho project, such leading Ibasho activities as opposed to only participating in daily and weekly events. Future studies can go deeper into how different elder groups with varying levels of social capital demonstrate different recovery experiences. Moreover, income can serve as an important source of resilience; however, this study measured income as the number of reported sources of funds, rather than actual amounts, due to many missing observations; therefore, the result of income needs to be reexamined in the future studies. Finally, this study focused on social capital and networks, but the results of this study also demonstrated that resilience is multidimensional and is associated with various social, economic, and cultural factors, such as age, education, gender, duration of residence, and income. Each of these factors can be critical in post-disaster recovery and should not be overlooked in future studies.

## Conclusion

Regression analysis as well as quasi-experimental propensity score matching and coarsened exact matching methods demonstrated stronger causal links between bottom-up, microlocal programs in post-shock areas and better recoveries. In a world facing increasing damage from natural hazards and extreme weather events, societies need ways to build resilience at the community level that can respond to a variety of shocks and work for people of all ages. As the critical nature of social capital becomes clear to scholars, disaster managers, and decision makers alike, more and more communities look to neighbor-focused interventions and microlocal programs to build cohesion, trust, and engagement^41^. But scholarship on the empirically measurable impact of these projects lags behind^42^. This study demonstrated that individuals participating in a community center-based project reported higher levels of family and neighborhood recovery than similar individuals who did not participate at all or as much. In addition, data also demonstrated the positive effects of social capital on disaster recovery.

Given the relatively low costs of social infrastructure such as community centers, libraries, and parks, disaster managers, local government officials, and residents of vulnerable communities should look to them as potential resources in building resilience to future shocks. Supporting qualitative studies of the role of social infrastructure^43^, this research provides solid quantitative evidence that investing in microlevel, community-tie building facilities simultaneously builds local cohesion while accelerating the pace of recovery after shocks. With societies worldwide facing more powerful natural hazards and simultaneously experiencing a growing population of elderly residents, projects such as Ibasho may be a model for building resilience.

## Data Availability

The dataset used during the current study is available in the Harvard Dataverse repository (https://doi.org/10.7910/DVN/A0AGMX).

## References

[1] Ngo EB (2001). When disasters and age collide: Reviewing vulnerability of the elderly. Nat. Hazards Rev..

[2] Heller K, Alexander DB, Gatz M, Knight BG, Rose T (2005). Social and personal factors as predictors of earthquake preparation: The role of support provision, network discussion, negative affect, age, and education. J. Appl. Soc. Psychol..

[3] Geller A (2009). The susceptibility of older adults to environmental hazards. Generations.

[4] Malak MA, Sajib AM, Quader MA, Anjum H (2020). “We are feeling older than our age”: Vulnerability and adaptive strategies of aging people to cyclones in coastal Bangladesh. Int. J. Disaster Risk Reduct..

[5] Bolin R, Klenow DJ (1983). Response of the elderly to disaster: An age-stratified analysis. Int. J. Aging Hum. Dev..

[6] Jia Z, Tian W, Liu W, Cao Y, Yan J, Shun Z (2010). Are the elderly more vulnerable to psychological impact of natural disaster? A population-based survey of adult survivors of the 2008 Sichuan earthquake. BMC Public Health.

[7] Meyer MA (2017). Elderly perceptions of social capital and age-related disaster vulnerability. Disaster Med. Public Health Prep..

[8] Beggs JJ, Haines VA, Hurlbert JS (1996). Situational contingencies surrounding the receipt of informal support. Soc. Forces..

[9] Dynes R (2005). Community Social Capital as the Primary Basis for Resilience.

[10] Pronyk PM (2008). Can social capital be intentionally generated? A randomized trial from rural South Africa. Soc. Sci. Med..

[11] Brune NE, Bossert T (2009). Building social capital in post-conflict communities: Evidence from Nicaragua. Soc. Sci. Med..

[12] Kiyota, E., Tanaka, Y., Arnold, M., & Aldrich, D. P. *Elders Leading the Way to Resilience*. (World Bank Group, Global Facility for Disaster Reduction and Recovery, 2016).

[13] Aldrich DP, Kiyota E (2017). Creating community resilience through elder-led physical and social infrastructure. Disaster Med. Public Health Prep..

[14] Haas, J. E., Kates, R. W., Bowden, M. J. *Reconstruction Following Disaster* (MIT Press, 1977).

[15] Rubin CB (2009). Long term recovery from disasters: The neglected component of emergency management. J. Homel. Secur. Emerg..

[16] Aldrich DP, Meyer MA (2015). Social capital and community resilience. Am. Behav. Sci..

[17] Rosenbaum PR, Rubin DB (1983). The central role of the propensity score in observational studies for causal effects. Biometrika.

[18] King G, Nielsen R (2019). Why propensity scores should not be used for matching. Polit. Anal..

[19] Brockie L, Miller E (2017). Understanding older adults’ resilience during the Brisbane floods: Social capital, life experience, and optimism. Disaster Med. Public Health Prep..

[20] Mayhorn CB (2005). Cognitive aging and the processing of hazard information and disaster warnings. Nat. Hazards Rev..

[21] Fernandez LS, Byard D, Lin CC, Benson S, Barbera JA (2002). Frail elderly as disaster victims: Emergency management strategies. Prehosp. Disaster Med..

[22] Gladwin H. & Peacock, W. G. Warning and evacuation: A night for hard houses. In *Hurricane Andrew: Ethnicity, Gender, and the Sociology of Disasters* (eds. Peacock, W. G., Morrow, B. H., & Gladwin, H.) 52–74 (Routledge, 1997).

[23] Aldrich DP, Sawada Y (2015). The physical and social determinants of mortality in the 3.11 Tsunami. Soc. Sci. Med..

[24] Wickes R, Zahnow R, Taylor M, Piquero AR (2015). Neighborhood structure, social capital, and community resilience: Longitudinal evidence from the 2011 Brisbane flood disaster. Soc. Sci. Q..

[25] Aldrich, D. P. *Black Wave: How Networks and Governance Shaped Japan’s 3/11 Disasters*. (University of Chicago Press, 2019).

[26] Manyena B, O'Brien G, O'Keefe P, Rose J (2011). Disaster resilience: A bounce back or bounce forward ability?. Local Environ..

[27] Adeola, F. O., Picou, J. S. Race, social capital, and the health impacts of Katrina: Evidence from the Louisiana and Mississippi Gulf Coast. *Hum. Ecol. Rev*. 10–24 (2012).

[28] Nakagawa Y, Shaw R (2004). Social capital: A missing link to disaster recovery. Int. J. Mass Emerg. Disasters..

[29] Lee J (2022). Voluntary associations and hazard preparedness behavior amongst Taiwanese individuals. Environ. Hazards..

[30] Lee J, Cho S (2018). The impact of crime rate, experience of crime, and fear of crime on residents’ participation in association: Studying 25 districts in the City of Seoul, South Korea. Crime Prev. Community Saf..

[31] Rayamajhee V, Bohara AK (2021). Social capital, trust, and collective action in post-earthquake Nepal. Nat. Hazards..

[32] Lee J (2022). Preparedness behaviors for natural hazards and their association with experiences, perceptions, and social engagement in Taiwanese society. Environ. Sociol..

[33] Lee J (2020). Bonding and bridging social capital and their associations with self-evaluated community resilience: A comparative study of East Asia. J. Community Appl. Soc. Psychol..

[34] Iwasaki K, Sawada Y, Aldrich DP (2017). Social capital as a shield against anxiety among displaced residents from Fukushima. Nat. Hazards..

[35] Panagopoulos C, Fraser T, Aldrich DP, Kim D, Hummel D (2021). Bridging the divide: Does social capital moderate the impact of polarization on health?. Polit. Res. Q..

[36] Brant R (1990). Assessing proportionality in the proportional odds model for ordinal logistic regression. Biometrics.

[37] Abadie A, Cattaneo MD (2018). Econometric methods for program evaluation. Annu. Rev. Econ..

[38] Olmos A, Govindasamy P (2015). A practical guide for using propensity score weighting in *R*. Pract. Assess. Res. Eval..

[39] Thoemmes FJ, Kim ES (2011). A systematic review of propensity score methods in the social sciences. Multivar. Behav. Res..

[40] Aldrich DP (2012). Building Resilience: Social Capital in Post-Disaster Recovery.

[41] Homsey, D. & Aldrich, D. P. Neighborfest: Building a stronger, more connected world from the block up. *Medium* https://medium.com/nextdooragencyresources/neighborfest-fc6fcd90f6b8. Accessed 20 April 2022 (2017).

[42] Villalonga-Olives E, Wind TR, Kawachi I (2018). Social capital interventions in public health: A systematic review. Soc. Sci. Med..

[43] Klinenberg, E. *Palaces for the People* (University of Chicago Press, 2018).

